# Parental Intention for Health Promotion in Children with Disabilities: Extending the Theory of Planned Behavior with Parenting Stress

**DOI:** 10.3390/children12121593

**Published:** 2025-11-24

**Authors:** Jinwoo Park, Seungho Ryu

**Affiliations:** 1 Department of Physical Education, Pusan National University, Busan 46241, Republic of Korea; wave3305@pusan.ac.kr; 2 Department of Sport Science, Pusan National University, Busan 46241, Republic of Korea

**Keywords:** parenting stress, extended theory of planned behavior, parental intention, health promotion, children with disabilities

## Abstract

**Highlights:**

**What are the main findings?**

- Parenting stress affects parental intention mainly via subjective norms and per-ceived behavioral control, with attitude having a limited role.
- Child-related stress impacts intention through attitude, while parent-related stress acts through social expectations and self-efficacy.

**What are the implications of the main findings?**

- Programs should strengthen parents’ capability and social support rather than focus only on attitudes.
- Reducing barriers, fostering networks, and using action planning can sustain long-term participation.

**Abstract:**

**Background/Objectives**: This study applies an Extended Theory of Planned Behavior, integrating parenting stress to examine factors influencing parental intentions to participate in health promotion programs for children with disabilities. **Methods**: Data (*n* = 345) were collected and analyzed using Structural Equation Modeling. **Results**: Results indicated that parenting stress affects intention indirectly through subjective norms and perceived behavioral control, with attitude playing a limited mediating role. Significant indirect effects were found from the child domain via attitude, and from the parent domain via both subjective norms and perceived behavioral control, while the sequential double-mediation pathway was not significant. **Conclusions**: These findings highlight the importance of addressing both social expectations and perceived capability in program design, alongside strategies to reduce structural barriers and enhance psychological and social support. Implications for culturally responsive interventions and the integration of action planning are presented.

## 1. Introduction

Since the enactment of Article 21 of the Act on Welfare Support for Children with Disabilities in Korea, developmental rehabilitation services designed to improve the functional abilities and treatment outcomes of children with disabilities have expanded significantly, particularly within private-sector and market-driven contexts [1]. These specialized services—including speech therapy, art therapy, play therapy, and, notably, adapted physical education—have become critical resources for families by offering structured opportunities for children to enhance their physical, cognitive, and social skills, thereby increasing participation in daily activities and improving overall quality of life. Such progress not only enables children with disabilities to engage more actively in physical activities but also allows families to spend more meaningful time together, share positive experiences, and reduce the caregiving burden through coordinated professional support. Consequently, these services now constitute a substantial portion of rehabilitation interventions in Korea, serving as both a therapeutic and a family-strengthening resource [2,3]. Within this landscape, active parental involvement is essential, particularly in light of increasing parental interest and expectations regarding the effectiveness of treatment and the role of adapted physical education in promoting overall health [4]. Despite the expansion of developmental rehabilitation services, participation in structured health promotion programs among children with disabilities remains substantially low. Parents often serve as the primary gatekeepers who determine whether their children can access such programs, making their participation intentions a critical determinant of service utilization.

Parental involvement typically entails active engagement in educational and therapeutic activities, as well as collaboration with professionals to maximize developmental outcomes for children [5]. When parents take an active role in their child’s education and daily life, it can significantly enhance language development, emotional adjustment, and academic achievement. For instance, research has shown that for children with autism spectrum disorder (ASD), parental efforts to establish structured and supportive home environments, utilize assistive communication tools, and collaborate consistently with educators are associated with improved learning outcomes and greater socio-emotional stability [6]. Similarly, for children with special educational needs, sustained parental advocacy has been linked to better access to appropriate resources and more favorable developmental trajectories [7]. Such involvement is especially crucial for children with disabilities, who often face significant challenges in communication, self-expression, and self-advocacy. These circumstances necessitate greater and more sustained parental engagement compared to that required for typically developing children [8].

Despite the well-recognized benefits of parental involvement, parents of children with disabilities frequently encounter substantial barriers, with parenting stress emerging as a particularly prominent challenge. Parenting stress refers to the emotional strain and psychological burden arising from intensive caregiving responsibilities, educational demands, financial pressures related to therapy, and persistent concerns about a child’s future [9,10]. Empirical studies consistently show that parents of children with disabilities experience stress levels two to three times higher than those of parents of typically developing children [4,10,11].

Elevated parenting stress can lead to parental burnout, characterized by physical, emotional, and psychological exhaustion, and may result in maladaptive parenting behaviors [11,12,13]. Consequently, interventions that reduce parenting stress are critical, as they foster positive parental attitudes, encourage proactive caregiving, and ultimately improve developmental outcomes for children [9,11,14]. Recent literature highlights the value of targeted stress-management programs and tailored interventions that address the unique stressors faced by these parents [11,15,16]. Nevertheless, despite the substantial body of research on parenting stress and its consequences, there remains a notable gap in systematically embedding these insights into theoretical frameworks that explain parental participation in health promotion programs for children with disabilities.

The Theory of Planned Behavior (TPB), proposed by Ajzen [17], predicts behavioral intentions and actions based on attitudes, subjective norms, and perceived behavioral control [18]. In recent years, TPB has been applied to a variety of health-related behaviors, including parental decision-making regarding health promotion and caregiving [19,20,21]. However, TPB alone does not fully capture the complex emotional and environmental challenges encountered by parents of children with disabilities. Consequently, recent studies have expanded TPB by incorporating additional contextual variables—such as parenting stress and action planning—to enhance its explanatory power and practical applicability [21,22,23].

Building on this work, the present study proposes an Extended Theory of Planned Behavior (ETPB) model that integrates parenting stress as a key determinant of parental intentions to support their child’s participation in health promotion programs. This theoretical extension offers practical implications for designing culturally responsive, evidence-based interventions aimed at promoting active parental involvement and improving health outcomes for children with disabilities. However, few studies have applied an extended TPB model specifically to parents of children with disabilities, and existing research has rarely integrated parenting stress into this theoretical framework.

Therefore, the aim of this study was to examine the factors influencing parental intentions using an extended TPB model incorporating parenting stress.

## 2. Materials and Methods

### 2.1. Participants

Participants in this study were parents of children with disabilities residing across 17 administrative regions in the Republic of Korea. Participants were recruited through child development centers. Center directors and therapists distributed paper-based questionnaires to eligible parents, and data were collected over a two-month period between September and October 2024.

Inclusion criteria were: (a) being a parent or primary caregiver of a child aged 3–18 with a documented disability, and (b) the ability to read and complete a Korean-language survey. Exclusion criteria included incomplete questionnaires or patterned/insincere responses. A total of 400 responses were obtained from parents registered at child development centers, of which 345 valid responses were retained after applying the exclusion criteria.

Among the participants, 99 (28.7%) were male and 246 (71.3%) were female. Regarding educational attainment, 59 parents (17.2%) held an associate degree, 247 (71.5%) a bachelor’s degree, and 39 (11.3%) a master’s degree or higher. Monthly household income levels were distributed as follows: less than $2000 (*n* = 10, 2.9%), $2000–2999 (*n* = 35, 10.2%), $3000–3999 (*n* = 141, 40.8%), $4000–4999 (*n* = 88, 25.6%), and $5000 or more (*n* = 71, 20.5%) (see Table 1).

The final sample size of 345 participants provides sufficient statistical power, exceeding the recommended minimum of 200 cases for the Monte Carlo technique [24]. This study complied with the Strengthening the Reporting of Observational Studies in Epidemiology (STROBE) guidelines. All participants were informed about the purpose and procedures of the study, and written informed consent was obtained prior to participation. Participation was voluntary, and respondents were assured of confidentiality and anonymity. An a priori power analysis using G*Power 3.1 indicated that a minimum of 200 participants is required to detect a medium effect size (f^2^ = 0.15) with 0.80 power at α = 0.05; therefore, our final sample of 345 participants satisfied the required statistical power.

### 2.2. Instruments

#### 2.2.1. Parenting Stress

To measure parental stress, Abidin et al. [19] multidimensional scale was adapted and modified. The parental stress analysis uses a 5-point Likert scale ranging from not at all 1 to very much 5, with a higher score indicating greater parental stress. The scale contains two dimensions—Child Domain (six items; e.g., “My child behaves in ways that are difficult to manage”) and Parent Domain (six items; e.g., “I feel emotionally drained due to parenting”). In the current study, the parental stress dimensions had nearly equivalent reliability coefficients (i.e., difficult child and parental distress Cronbach’s α = 0.80 and 0.79, respectively).

#### 2.2.2. Attitude

To measure attitude, Rimmerman & Chen’s [20] attitude scale was adapted and modified. The attitude (three items; e.g., “Supporting my child’s participation in health promotion programs is beneficial”) analysis uses a 5-point Likert scale ranging from not at all 1 to very much 5, with a higher score indicating higher level of attitude. In the current study, the attitude scale (i.e., Cronbach’s α = 0.73, respectively) had nearly equivalent reliability coefficients.

#### 2.2.3. Subjective Norm

To measure subjective norm, Rimmerman & Chen’s [20] subjective norm scale was adapted and modified. The subjective norm (three items; “People around me expect me to support my child’s participation”) analysis uses a 5-point Likert scale ranging from not at all 1 to very much 5, with a higher score indicating higher level of subjective norm. In the current study, the subjective norm scale (i.e., Cronbach’s α = 0.84, respectively) had nearly equivalent reliability coefficients.

#### 2.2.4. Perceived Behavioral Control

To measure perceived behavioral control, Rimmerman & Chen’s [20] perceived behavioral control scale was adapted and modified. The perceived behavioral control analysis (three items; “I feel capable of helping my child participate in such programs”) uses a 5-point Likert scale ranging from not at all 1 to very much 5, with a higher score indicating higher level of perceived behavioral control. In the current study, the perceived behavioral control scale (i.e., Cronbach’s α = 0.79, respectively) had nearly equivalent reliability coefficients.

#### 2.2.5. Parental Intention

To measure parental intention, Perry & Langley’s [21] parental intention scale was adapted and modified. The parental intention analysis (three items; e.g., “I intend to support my child’s participation in health promotion programs”) uses a 5-point Likert scale ranging from not at all 1 to very much 5, with a higher score indicating higher level of parental intentions. In the current study, the parental intentions scale (i.e., Cronbach’s α = 0.91, respectively) had nearly equivalent reliability coefficients.

All measurement items and reliability information are summarized in Table 2.

### 2.3. Data Analysis

Descriptive statistics, normality of variables, and correlations were first computed using the Statistical Package for the Social Sciences (SPSS) 29.0. The psychometric properties of the measurement model were assessed via Confirmatory Factor Analysis (CFA) with maximum likelihood estimation using the AMOS 26.0 software. To examine the goodness of fit of the measurement model, the following fit indices and cut-off values were assessed [25]: normed chi-square *x*^2^/df, root mean square error of approximation (RMSEA; <0.08), standardized Root Mean Square Residual (SRMR; <0.08), and Comparative Fit Index (CFI; >0.90). After testing the measurement model, the Structural Equation Modeling (SEM) technique was employed to examine the relationship among the variables in ETPB also using the of AMOS 26.0.

## 3. Results

Table 3 presents the Pearson product–moment correlations, means, and standard deviations for the study variables. The analysis of the relationships among these variables revealed correlation coefficients ranging from weak to moderate (0.15–0.40), with both positive and negative directions. Specifically, Child Domain was negatively correlated with Attitude (*r* = −0.15, *p* < 0.01), while Parent Domain showed a significant positive correlation with Child Domain (*r* = 0.25, *p* < 0.01) and significant negative correlations with Subjective Norm (*r* = −0.21, *p* < 0.01), Perceived Behavioral Control (*r* = −0.22, *p* < 0.01), and Parental Intention (*r* = −0.15, *p* < 0.01). Attitude was positively correlated with Subjective Norm (*r* = 0.24, *p* < 0.01) and Perceived Behavioral Control (*r* = 0.17, *p* < 0.01), but not significantly correlated with Parental Intention. Subjective Norm demonstrated significant positive correlations with Perceived Behavioral Control (*r* = 0.40, *p* < 0.01) and Parental Intention (*r* = 0.33, *p* < 0.01), and Perceived Behavioral Control was also positively correlated with Parental Intention (*r* = 0.27, *p* < 0.01).

Confirmatory factor analysis supported the adequacy of the six-factor measurement model (*x*^2^/df = 2.56, RMSEA = 0.06, IFI = 0.93, TLI = 0.91, CFI = 0.93, SRMR = 0.05). The hypothesized structural equation model demonstrated acceptable fit to the data (*x*^2^/df = 2.97, RMSEA = 0.07, IFI = 0.91, TLI = 0.90, CFI = 0.91, SRMR = 0.07).

Path analysis revealed several significant direct effects. Child Domain negatively predicted Attitude (*β* = −0.12, *p* < 0.01) but had no significant effects on Subjective Norm (*β* = 0.05, *p* > 0.05) or Perceived Behavioral Control (*β* = 0.07, *p* > 0.05). Parent Domain negatively predicted Subjective Norm (*β* = −0.17, *p* < 0.01) and Perceived Behavioral Control (*β* = −0.20, *p* < 0.01) but was not significantly associated with Attitude (*β* = 0.05, *p* > 0.05). Subjective Norm (*β* = 0.48, *p* < 0.01) and Perceived Behavioral Control (*β* = 0.31, *p* < 0.01) positively predicted Parental Intention, whereas Attitude did not show a significant direct effect (*β* = 0.01, *p* > 0.05).

Bootstrapping analysis (*n* = 5000, bias-corrected) indicated multiple significant indirect effects. Child Domain had a significant indirect effect on Parental Intention via Attitude (*β* = 0.24, 95% CI [0.04, 0.15], *p* < 0.01), but indirect effects through Subjective Norm or Perceived Behavioral Control were not significant. Parent Domain had significant indirect effects on Parental Intention through Subjective Norm (*β* = 0.36, 95% CI [0.08, 0.26], *p* < 0.01) and through Perceived Behavioral Control (*β* = 0.22, 95% CI [0.12, 0.13], *p* < 0.01).

**Figure 1:**
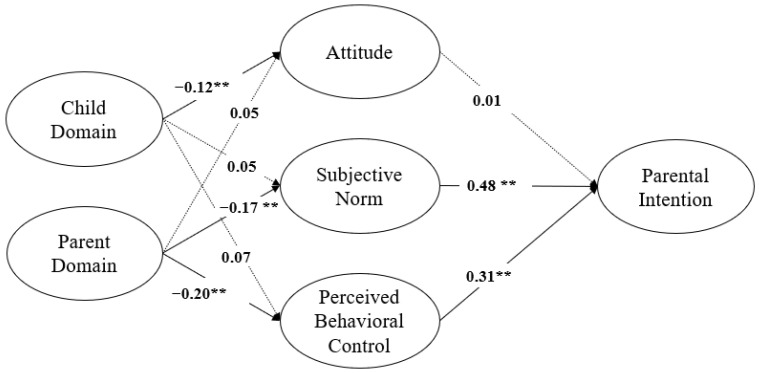
The results of the structural equation modeling. Arrows indicate the directional relationships among the constructs. ** *p* < 0.01.

Overall, these findings suggest that the influence of parenting stress on parental intention operates primarily through social expectations and perceived capability, with attitudinal change playing a limited mediating role in the model (Figure 1).

## 4. Discussion

This study employed an Extended Theory of Planned Behavior (ETPB) incorporating parenting stress to explore factors influencing parental intentions to participate in health promotion programs for children with disabilities. Structural Equation Modeling (SEM) findings supported the proposed model and clarified how different dimensions of parenting stress are linked to key TPB constructs.

The child domain of parenting stress was associated with lower parental attitudes, aligning with the significant negative relationship observed between the two variables as reflected in both the correlation results (*r* = −0.15, *p* < 0.01) and the structural path estimate (*β* = −0.12, *p* < 0.01). This pattern is consistent with previous research showing that challenging child behaviors are linked to increased parental burden and less favorable evaluations of intervention efforts [11,22]. In this study, the association between the child domain and parental intention emerged primarily through attitude, corresponding to the significant indirect pathway identified in the bootstrapping analysis, suggesting that attitudinal shifts represent the primary pathway linking child-related stress to intention. International research demonstrates similar TPB extensions in Europe, North America, and Asia, where parental stress influences health-related decision-making primarily through perceived behavioral control and social support [21,22,23,26].

In contrast, the parent domain of stress demonstrated a broader pattern of associations. Parent-related stress was linked to lower subjective norms and perceived behavioral control, consistent with the observed negative relationships in the correlation and structural analyses (Subjective Norm: *r* = −0.21, *β* = −0.17; Perceived Behavioral Control: *r* = −0.22, *β* = −0.20; all *p* < 0.01). These findings resonate with earlier work showing that heightened parental stress is associated with reduced self-efficacy and less proactive engagement in supportive behaviors [11,22]. In the present study, the parent domain also showed indirect associations with parental intention through subjective norm and perceived behavioral control, reflecting the significant indirect effects detected in the mediation analysis, indicating that these social and capability-related mechanisms may play an important role in explaining intention formation under conditions of elevated parenting stress.

Interestingly, subjective norms and perceived behavioral control were positively associated with parental intention, supported by both the correlation matrix (Subjective Norm: *r* = 0.33; Perceived Behavioral Control: *r* = 0.27) and the structural model (*β* = 0.48 and *β* = 0.31, respectively; *p* < 0.01). In contrast, attitude showed no direct effect on intention, consistent with the nonsignificant correlation (*r* = 0.08) and structural path (*β* = 0.01, *p* > 0.05). This diverges from conventional TPB applications, where attitude typically plays a central predictive role [21,23]. The non-significant effect of attitude may be explained by stress acting as a moderator that weakens the attitude–intention link, consistent with findings reported by Hamilton et al. [26]. In high-stress caregiving contexts, however, practical considerations—such as capability and perceived social support—may carry more weight than internal evaluations in shaping behavioral intentions [23]. Recent evidence by Hsiao [27] demonstrated that higher family resilience mitigates the adverse effects of parenting stress on health-related quality of life in parents of children with disabilities, suggesting that resilience-enhancing interventions may help buffer stress impacts on TPB constructs.

The relatively limited role of subjective norms in this study contrasts with findings from Durmus and Sarol [28], who emphasized the influence of professional guidance and social approval. This may reflect cultural differences, as Korean parents might prioritize personal judgment and firsthand experience over external recommendations. This underscores the need for culturally responsive interventions that respect parental autonomy [3,4,11]. Moreover, Lu et al. [29] found that social support both mediates and moderates the relationship between parenting stress and life satisfaction among parents of children with autism spectrum disorder, reinforcing the importance of integrating strong support networks into intervention design.

Drawing on the work of Ku and Haegele [22], the present findings also highlight the potential of action planning as a bridge between intention and behavior. Action planning—encompassing goal setting, structured scheduling, and coping strategies—may be particularly useful for parents facing high caregiving demands. This view aligns with meta-analytic evidence from Hamilton et al. [26], which shows that extended TPB models incorporating planning constructs significantly improve behavioral prediction and help bridge the intention–behavior gap.

The findings carry practical implications. Policies and programs should aim to reduce structural barriers such as time constraints, economic limitations, and accessibility challenges. Tailored services that offer flexibility, affordability, and ease of access are critical to supporting parental involvement [11,23]. In parallel, psychological support strategies—including stress management programs, peer support groups, and parent education—should be implemented to strengthen parents’ emotional resilience and sense of agency [11,22,27,29].

Future research should investigate the mechanisms underlying the limited influence of subjective norms through qualitative inquiry and cross-cultural comparisons. Furthermore, longitudinal designs are needed to evaluate both the long-term effectiveness of incorporating action planning into health promotion interventions for families of children with disabilities [22,23,26] and to address the limitations associated with convenience sampling and a cross-sectional research design, which restrict causal inference and generalizability.

## 5. Conclusions

This study extends the traditional Theory of Planned Behavior (TPB) by integrating parenting stress to explain parental intentions to support their child’s participation in health promotion programs. The results highlight that parenting stress influences behavioral intentions primarily through subjective norms and perceived behavioral control, underscoring the need to address these factors in program design.

From a practical standpoint, interventions should move beyond solely attitude-focused approaches and adopt comprehensive, context-sensitive strategies that alleviate structural barriers—such as time constraints, economic burdens, and limited service accessibility—while providing psychological support (e.g., stress management and resilience training) and robust social support networks. Culturally responsive approaches that respect parental autonomy, strengthen peer-parent networks, foster parent–professional collaboration, and adopt flexible service delivery models can enhance perceived behavioral control and sustain long-term participation. Furthermore, incorporating action planning—encompassing goal setting, structured scheduling, and coping strategies—offers a promising means to translate intentions into sustained behavioral change, as supported by evidence from extended TPB models [26].

## Figures and Tables

**Table 1:** Demographic and socioeconomic characteristics of participants.

Characteristic	Category	n	%
Gender of parent	Male	99	28.7
	Female	246	71.3
Education level	Associate degree	59	17.2
	Bachelor’s degree	247	71.5
	Master’s degree or higher	39	11.3
Monthly household income	<$2000	10	2.9
	$2000~$2999	35	10.2
	$3000~$3999	141	40.8
	$4000~$4999	88	25.6
	≥$5000	71	20.5

**Table 2:** Summary of Cronbach’s Alpha, CR, AVE Values.

Factors/Items	λ	α	CR	AVE
Child Domain		0.80	0.93	0.70
My child behaves in ways that are difficult to manage.	0.77			
I find it challenging to discipline my child effectively.	0.71			
My child often ignores rules or instructions.	0.87			
My child requires more supervision than others.	0.78			
My child’s behavior often frustrates me.	0.82			
My child’s demands are exhausting.	0.81			
Parent Domain		0.79	0.94	0.73
I feel overwhelmed by my responsibilities as a parent.	0.79			
I feel tired and worn out from parenting.	0.74			
I feel emotionally drained due to parenting demands.	0.78			
I feel I am not doing a good job as a parent.	0.81			
I worry a lot about my child’s future.	0.86			
Parenting makes me feel anxious and depressed.	0.79			
Attitude		0.73	0.94	0.83
*“Taking an interest in and being involved in my child’s participation in health promotion programs is…”*				
Helpful for my child	0.98			
Worthwhile	0.88			
A positive action	0.93			
Subjective Norm		0.84	0.93	0.82
*“People around me think I should take an interest in and be involved in my child’s participation in health promotion programs…”*				
My family supports my involvement.	0.83			
My friends and acquaintances value my interest and support.	0.79			
Professionals (e.g., therapists, teachers) expect me to be involved.	0.87			
Perceived Behavioral Control		0.79	0.91	0.79
*“When it comes to taking an interest in and being involved in my child’s participation in health promotion programs…”*				
I have sufficient information and resources.	0.79			
I am capable of adjusting my efforts to support my child.	0.76			
I feel able to overcome barriers and assist my child effectively.	0.87			
Parental Intention		0.91	0.93	0.81
*“I intend to take an active interest in and be involved in my child’s participation in health promotion programs…”*				
I will continue to stay informed and engaged.	0.86			
I plan to seek out information and follow up on participation opportunities.	0.84			
I am willing to help my child participate actively whenever possible	0.81			

Note. λ = standardized factor loading; α = Cronbach’s alpha; CR = composite reliability; AVE = average variance extracted.

**Table 3:** Correlations, means, and SD among parenting stress and TPB.

Scales		1	2	3	4	5	6
1.	Child Domain						
2.	Parent Domain	0.25 **					
3.	Attitude	−0.15 **	0.01				
4.	Subjective Norm	−0.01	−0.21 **	0.24 **			
5.	Perceived Behavioral Control	0.01	−0.22 **	0.17 **	0.40 **		
6.	Parental Intention	0.01	−0.15 **	0.08	0.33 **	0.27 **	
	*M*	2.91	1.97	4.59	4.54	4.54	4.49
	*SD*	0.79	0.81	0.45	0.50	0.49	0.58

** *p* < 0.01.

## Data Availability

The data presented in this study are not publicly available due to privacy and ethical restrictions related to sensitive information from participants. However, the data may be made available from the corresponding author upon reasonable request and with permission from the Institutional Review Board.
